# Development of Asthma-Like Symptoms After COVID-19: A Cross-Sectional Study in Dubai, United Arab Emirates

**DOI:** 10.7759/cureus.48591

**Published:** 2023-11-09

**Authors:** Syed Arshad Husain, Amirarshia Rategh, Muhammad Omar Larik, Leon G D'Cruz, Jean Mary John, Bassam Mahboub

**Affiliations:** 1 Department of Pulmonology, King's College Hospital London, Dubai, ARE; 2 Faculty of Science, University of British Columbia, Vancouver, CAN; 3 Department of Medicine, Dow International Medical College, Karachi, PAK; 4 Department of Research, Portsmouth Hospitals NHS Trust, Portsmouth, GBR; 5 Department of Pulmonology, Rashid Hospital, Dubai, ARE

**Keywords:** respiratory system, post-covid effects, post-covid asthma, coronavirus, covid-19, asthma

## Abstract

Background

Coronaviruses are viral agents that commonly infect animals, but have the ability to cause respiratory illness in humans, exemplified by the ongoing novel coronavirus outbreak (COVID-19). Due to the sparse literature on the effects of COVID-19 on the respiratory system, and the possible development of persistent asthma-like symptoms after infection, this cross-sectional analysis was performed in order to compare the clinical and investigative parameters between post-COVID patients and asthmatic patients.

Methods

A retrospective cross-sectional study was conducted on patients with prior history of COVID-19 infection that presented to the pulmonology or respiratory outpatient clinics with asthma-like symptoms and were subsequently compared to known asthmatic patients with absent history of prior COVID-19 infection, in order to evaluate the degree of similarity between both cohorts. In this study, asthma-like symptoms were defined as: (i) cough, (ii) wheezing, (iii) chest tightness, and (iv) shortness of breath. Moreover, comparisons of investigative parameters were also performed, including (i) fractional exhaled nitric oxide (FeNO), (ii) serum immunoglobulin E (IgE), (iii) absolute eosinophil counts, and (iv) qualitative spirometry results. All statistical analyses were conducted via chi-squared testing for categorical variables, and independent t-test for continuous variables.

Results

In this study, there were a total of 76 patients included that conformed to the eligibility criteria, including 39 patients with post-COVID symptoms with absent history of asthma or other respiratory illnesses, and 37 patients with known asthma with absent history of prior COVID-19 infection or other respiratory illnesses. Overall, this study revealed the similarities between both cohorts with respect to the incidence of cough, chest tightness, and shortness of breath. Moreover, there were similarities between the serum IgE and spirometry results. However, there were differences within the complaint of wheeze, FeNO values, and eosinophil counts between both cohorts. The placement of post-COVID patients on bronchodilator therapy involving inhaled corticosteroids and long-acting beta-agonists revealed improvement in all follow-up patients.

Conclusion

In conclusion, there was considerable similarity in the complaint of asthma-like symptoms after COVID-19 infection, associated with an improvement after the use of bronchodilator therapy, indicating the potential role of anti-asthma therapy (e.g., bronchodilator therapy) in managing post-COVID asthma-like symptoms. In order to validate our conclusion, further comprehensive studies with robust methodologies and larger sample populations are encouraged.

## Introduction

Coronaviruses are a group of viral agents that commonly infect animals, although possess the ability to cause respiratory illnesses in humans [1]. This is not the first outbreak that has arisen from this virus, as this zoonotic pathogen has caused severe acute respiratory syndrome (SARS-CoV) and the Middle East respiratory syndrome coronavirus (MERS-CoV), which occurred in 2002 and 2012, respectively [2]. Towards the end of 2019, the notorious viral agent demonstrated re-emergence from Wuhan, China, and significantly surpassed the infectivity and lethality of the existing SARS-CoV and MERS-CoV strains. Thus, the novel coronavirus disease 2019 (COVID-19) had been declared as a major global threat [3].

COVID-19 has been associated with poorer prognosis within certain populations, particularly those individuals who are suffering from pre-existing conditions, such as asthma and chronic obstructive pulmonary disease (COPD). Recent studies have revealed the heightened risk of developing severe COVID-19 disease in patients with COPD, despite patient-level adjustments for age and smoking [4,5]. There are several speculations that shed light on the background mechanism behind the apparent increased risk of disease severity within COPD patients. COVID-19 virus enters the cell via the angiotensin-converting enzyme-2 (ACE-2) receptor, and the ACE-2 expression was found to be drastically elevated in COPD patients, but also among smokers and frequent nicotine users [6-8]. Thus, the presentation of post-COVID patients in pulmonology clinics with asthma-like symptoms has increased, which may be suggestive of the development of persistent symptoms after COVID-19 infection.

Due to the increasing significance of COVID-19 and the sparseness of existing published literature exploring the post-COVID syndrome, this single-center retrospective analysis was conducted in a tertiary-care hospital located in Dubai, United Arab Emirates, comparing the complaints of post-COVID asthma-like symptoms with those of existing patients with known type 2 asthma. Moreover, comparisons using investigative parameters, including fractional exhaled nitric oxide (FeNO), eosinophil concentrations, immunoglobulin E (IgE) values, and spirometry results were also performed within this study. This sample population serves to be an excellent choice due to the vast diversity and high density of expatriate populations residing within the region, giving indiscriminate results that may be applicable to other regions of the world. Moreover, within the timeframe of this study, most COVID-19 sufferers were either partially or fully vaccinated, leading to a less severe infection, and hence providing a new perspective on the outlook of the disease.

## Materials and methods

Study design

This retrospective cross-sectional study was conducted at the King’s College Hospital London, Dubai, United Arab Emirates, where patients were seen in the clinic between January 2022 to May 2023. The sample population consisted of patients visiting the outpatient department at the pulmonology clinic, with chief complaints of asthma-like symptoms, defined as cough, wheezing, chest tightness, and shortness of breath (SOB). Fully or partially vaccinated patients with both early post-COVID and late post-COVID symptoms were reviewed in this study, presenting a minimum of 2 weeks, and a maximum of 6 months after their initial COVID-19 diagnosis. Such patients had obtained a positive result via real-time polymerase chain reaction (RT-PCR) COVID-19 testing. Approval to conduct this study was sought from the Research and Ethics Committee at King’s College Hospital London, Dubai, United Arab Emirates (KCH/MOI/740).

Data collection and extraction

The electronic medical record system of the hospital was utilized for data collection. The following patient information was extracted for study purposes, including (i) age, (ii) gender, (iii) body mass index (BMI), (iv) COVID-19 history, and (v) complaint of asthma-like symptoms, including cough, wheeze, chest tightness, and SOB. Moreover, the following investigative parameters were extracted as part of the clinic’s standard investigation panel, including (i) FeNO levels, (ii) absolute eosinophil levels, (iii) serum IgE, and (iv) spirometry results. The post-COVID patients with asthma-like symptoms were placed on bronchodilators (inhaled corticosteroids + long-acting beta agonist) as per the standard practice of the clinic, and any relief of symptoms was evaluated on follow-up. For the purpose of comparisons performed within the study, the reference ranges for FeNO are declared at ≤25 ppb as normal, and >25 ppb as abnormal [9]. Similarly, the reference ranges for absolute eosinophil counts are declared at ≤1 x 10^9^/L as normal, and >1 x 10^9^/L as abnormal [10]. Additionally, the reference ranges for serum IgE are declared at ≤112 IU/mL as normal, and >112 IU/mL as abnormal [11]. The BMI ranges were categorized in accordance with the World Health Organization’s (WHO) classification, in which individuals under <18.5 kg/m^2^ were underweight, individuals between 18.5 to 24.9 kg/m^2^ were healthy, individuals between 25 to 29.9 kg/m^2^ were overweight, and individuals ≥30 kg/m^2^ were obese [12].

Statistical analyses

All statistical analyses were performed using Statistical Package for Social Software (SPSS) version 20.0 (IBM Corp, Armonk, New York, USA), with various statistical tests employed. The chi-squared test was utilized for the comparison of categorical variables of two cohorts. Additionally, the independent t-test was utilized for the comparison of continuous variables of two cohorts. Statistical significance was denoted at a p-value equal to or below 0.05. Two cohorts were created for this study, with (i) Group A representing post-COVID patients with absent history of asthma or any other underlying respiratory illness, and (i) Group B representing an asthmatic cohort with absent history of COVID-19 infection or any other underlying respiratory illness. Consequently, the asthma-like symptoms were compared between both cohorts. The lack of statistical significance between the two cohorts indicates a strong similarity between both cohorts, stipulating the development of post-COVID asthma-like symptoms in patients who were not known asthmatics prior to infection.

## Results

Demographic characteristics of the study population

In this retrospective study, there were a total of 76 patients included through the use of pre-specified eligibility criteria applied during the systematic review of the hospital database. Out of those 76, there were 39 patients included in Group A (post-COVID patients with no history of asthma or respiratory illness) and there were 37 patients included in Group B (known asthmatic patients). The median age of Group A patients was 42 years (IQR: 36-47) and the median age of Group B patients was 42 years (IQR: 35-55), with no significant differences between the ages of both cohorts (p = 0.45). There were 15 males and 24 females in Group A, and 18 males and 19 females in Group B, with no significant differences between the sexes of both cohorts (p = 0.37). The majority of Group A was overweight, with a mean BMI of 27.5 kg/m^2^ (SD: 6.6), and the majority of Group B was overweight, with a mean BMI of 27.2 kg/m^2^ (SD: 4.5), with no significant differences between the BMI of both cohorts (p = 0.79). The baseline demographic characteristics of the included patients have been illustrated in Table 1.

**Table 1:** Baseline demographic information of Group A (post-COVID patients) and Group B (asthmatic patients). n: number of participants; y: years; IQR: interquartile range; BMI: body mass index; SD: standard deviation.

Variable		Overall (n = 76)	Group A (n = 39)	Group B (n = 37)	P-value
Age	Median Age, y (IQR)	42 (36-51)	42 (36-47)	42 (35-55)	0.45
	<40 years, n (%)	34 (44.7)	17 (43.6)	17 (45.9)	
	≥40 years, n (%)	42 (55.3)	22 (56.4)	20 (54.1)	
Gender	Male, n (%)	33 (43.4)	15 (38.5)	18 (48.6)	0.37
	Female, n (%)	43 (56.6)	24 (61.5)	19 (51.4)	
BMI	Mean BMI, kg/m^2^ (SD)	27.3 (5.7)	27.5 (6.6)	27.2 (4.5)	0.79
	Underweight, n (%)	2 (2.6)	1 (2.6)	1 (2.7)	
	Healthy, n (%)	22 (28.9)	13 (33.3)	9 (24.3)	
	Overweight, n (%)	32 (42.2)	17 (43.6)	15 (40.6)	
	Obese, n (%)	20 (26.3)	8 (20.5)	12 (32.4)	

Comparison of asthma-like symptoms

**Figure 1:**
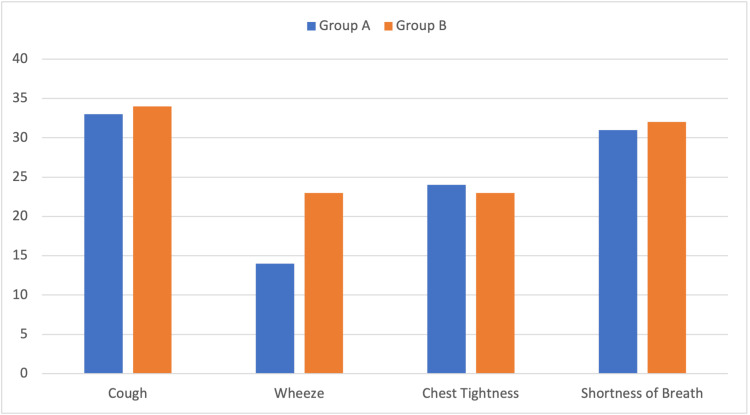
Comparison of Group A (post-COVID patients) and Group B (asthmatic patients) with respect to complaints of asthma-like symptoms.

The following asthma-like symptoms were compared between both cohorts, including: (i) cough, (ii) wheeze, (iii) chest tightness, and (iv) SOB. Overall, there were no significant differences observed in the symptom of cough between Group A and Group B (p = 0.33). Contrastingly, there were significant differences noted in the symptom of wheeze between Group A and Group B (p = 0.02). Additionally, there were no significant differences observed in the symptom of chest tightness between Group A and Group B (p = 0.96). Similarly, there were no significant differences observed in the symptom of SOB between Group A and Group B (p = 0.42). In summary, the incidence of symptoms of cough, chest tightness, and SOB was similar between both cohorts, indicating the possible development of post-COVID asthma-like symptoms. There was a symptomatic improvement observed within Group A after the commencement of the standard asthma treatment (inhaled corticosteroids + long-acting beta agonist). The summarized comparison of asthma-like symptoms is illustrated in Figure 1, and the detailed comparison of asthma-like symptoms is available in Table 2.

**Table 2:** Comparison of Group A (post-COVID patients) and Group B (asthmatic patients) with respect to complaints of asthma-like symptoms. n: number of patients.

Variable		Overall (n = 76)	Group A (n = 39)	Group B (n = 37)	P-value
Incidence of asthma-like symptoms	Cough, n (%)	67 (88.2)	33 (84.6)	34 (91.9)	0.33
	Wheeze, n (%)	37 (48.7)	14 (35.9)	23 (62.2)	0.02
	Chest tightness, n (%)	47 (61.8)	24 (61.5)	23 (62.2)	0.96
	Shortness of breath, n (%)	63 (82.9)	31 (79.5)	32 (86.5)	0.42
Treatment outcome after bronchodilator therapy	Improvement, n	24	24		
	No improvement, n	0	0		
	Lost to follow-up, n	15	15		

Comparison of investigative findings

**Figure 2:**
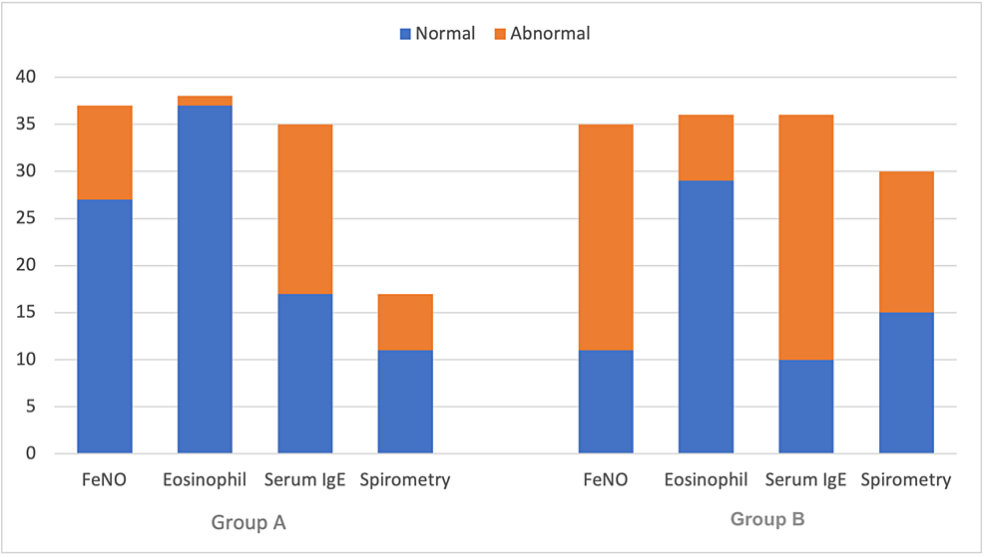
Comparison of Group A (post-COVID patients) and Group B (asthmatic patients) with respect to investigative parameters. FeNO: fractional exhaled nitric oxide; IgE: immunoglobulin E.

The following investigative parameters were compared between both cohorts, including (i) FeNO levels, (ii) absolute eosinophil counts, (iii) serum IgE levels, and (iv) qualitative spirometry results. Overall, there were significant differences observed in the FeNO levels between Group A and Group B (p < 0.0001), with the mean value of Group A equal to 26.2 ppb (SD: 25.8) compared with the mean value of Group B equal to 68.3 ppb (SD: 57.9). Similarly, there were significant differences observed in the eosinophil counts between Group A and Group B (p = 0.004), with the mean value of Group A equal to 0.19x10^9^/L (SD: 0.15) compared with the mean value of Group B equal to 0.42x10^9^/L (SD: 0.44). Contrastingly, there were no significant differences observed in the serum IgE levels between Group A and Group B (p = 0.40), with the mean value of Group A equal to 346 IU/mL (SD: 583) compared with the mean value of Group B equal to 457.8 IU/mL (SD: 482). The most common spirometry presentations included normal (34.2%), followed by obstructive (18.4%), with no significant differences observed within the spirometry results between Group A and Group B (p = 0.10). The summarized comparison of asthma-like symptoms is illustrated in Figure 2, and the detailed comparison of asthma-like symptoms is available in Table 3.

**Table 3:** Comparison of Group A (post-COVID patients) and Group B (asthmatic patients) with respect to investigative parameters. n: number of patients; SD: standard deviation; FeNO: fractional exhaled nitric oxide; IgE: immunoglobulin E.

Variable		Overall (n = 76)	Group A (n = 39)	Group B (n = 37)	P-value
FeNO	Mean FeNO, ppb (SD)	46.7 (48.9)	26.2 (25.8)	68.3 (57.9)	< 0.0001
	Normal, n (%)	38 (50)	27 (69.2)	11 (29.7)	
	Abnormal, n (%)	34 (44.7)	10 (25.6)	24 (64.9)	
	Not reported, n (%)	4 (5.3)	2 (5.2)	2 (5.4)	
Eosinophil	Mean Eosinophil Count, x10^9^/L (SD)	0.29 (0.34)	0.19 (0.15)	0.42 (0.44)	0.004
	Normal, n (%)	66 (86.8)	37 (94.8)	29 (78.4)	
	Abnormal, n (%)	8 (10.6)	1 (2.6)	7 (18.9)	
	Not reported, n (%)	2 (2.6)	1 (2.6)	1 (2.7)	
IgE	Mean IgE, IU/mL (SD)	400 (450)	346 (584)	458 (482)	0.40
	Normal, n (%)	27 (35.5)	17 (43.6)	10 (27.0)	
	Abnormal, n (%)	44 (57.9)	18 (46.2)	26 (70.3)	
	Not reported, n (%)	5 (6.6)	4 (10.2)	1 (2.7)	
Spirometry	Normal, n (%)	26 (34.2)	11 (28.2)	15 (40.5)	0.10
	Obstructive, n (%)	14 (18.4)	2 (5.1)	12 (32.5)	
	Restrictive, n (%)	7 (9.2)	4 (10.3)	3 (8.1)	
	Not reported, n (%)	29 (38.2)	22 (56.4)	7 (18.9)	

## Discussion

This retrospective analysis revealed that post-COVID patients, in the absence of known asthma, presented with similar symptoms of cough, chest tightness, and SOB when compared to type 2 asthmatic patients. Such patients were included in this study in the absence of any known pneumonitis or interstitial lung disease, which leads to COVID-19 being the presumable etiological agent for the development of this “syndrome” of symptoms that were not present before. To the best of our knowledge, this is the first study of this nature within the Middle Eastern region, with the United Arab Emirates featuring a diversified population, and an exceptional COVID-19 vaccination rate nearing 100%. Thus, this provides novel preliminary data in a population where severe COVID-19 infection is uncommon, revealing a new perspective on the development of post-COVID asthma-like symptoms.

In a comprehensive analysis performed on data from the United Kingdom’s Biobank, it was observed that the asthmatic population faced an apparent protection against COVID-19 infection; however, this observation was only significant for patients under the age of 65 [13]. Contrastingly, the presence of asthma acted as a risk factor for COVID-19 hospitalization, indicating a potentially complex mechanism behind these findings. The concept of post-COVID complications has been established in other systems [14,15]. In a study conducted by Huang et al. in China, it was observed that hospitalized patients experienced persistent symptoms and abnormalities in cardiovascular and lung function, such as fatigue, SOB, chest pain, and evidence of myocarditis in some cases [14]. In another article written by Kanne et al., it was concluded that one in three COVID-19 pneumonia patients reported some form of long-term complications as per their chest computed tomography (CT) scans performed one year after discharge [16]. Such abnormalities included parenchymal bands, frank fibrosis, and bronchial dilation. Overall, this study highlighted the possibility of post-infectious respiratory sequelae, whilst also stating how previous similar studies faced certain limitations, ranging from smaller sample sizes to the possibility of information bias, indicating strong validity within their results. Additionally, in a similar study conducted within the pediatric population post-COVID hospitalization, it was deduced that there was an increased prevalence of asthma-like symptoms in such children, particularly in those with a strong family history of allergic rhinitis [17].

Furthermore, in a study conducted by Lior et al., it was suggested that low FeNO measurements could potentially indicate susceptibility to respiratory complications of COVID-19, highlighting an opportunity for the use of FeNO values as a prognostic indicator for long-term severity of complications, especially in resource-limited settings within developing countries [18]. Furthermore, the use of inflammatory markers may also be useful in providing prognostic information (including C-reactive protein and lymphocytes) by predicting the risk of hospitalization during COVID-19 infection [19,20]. Thus, further research delving into the use of different biomarkers to diagnose post-COVID asthma is warranted.

It is important to note that testing alone is insufficient for diagnosing or managing the potential development of post-COVID asthma-like symptoms, and should be used in conjunction with thorough and comprehensive clinical evaluations performed by healthcare specialists. Further research is needed in order to fully understand the impact of COVID-19 on the respiratory system, and how post-COVID patients can be managed, who suffer from asthma-like symptoms. In a systematic review and meta-analysis conducted by Griesel et al., it was reported that mild COVID-19 infection can be managed with inhaled corticosteroids, thus indicating the potential role of inhalers in providing benefit for post-COVID asthma-like symptoms, as observed within our study [21].

Future recommendations

Although our study preliminarily demonstrated excellent short-term treatment outcomes involving inhalers, it is still important to acknowledge the question of whether these symptoms will reoccur after initial treatment, or require bronchodilator therapy in the future. Therefore, there is a valid necessity to conduct further research on the possible long-term persistence and subsequent long-term management strategies of post-COVID asthma-like symptoms in a longitudinal setting. Moreover, it is important to explore how potential biomarkers or investigations may be utilized to monitor and manage such patients. The establishment of the correlation of post-COVID asthma-like symptoms in severe cases has various clinical implications that must be acclaimed. This requires the frequent, long-term monitoring of such patients after their infection, potentially through biomarkers mentioned previously, or various other investigative tests such as FeNO or spirometry. In cases of post-COVID asthma-like symptoms, such patients can be placed on the standard treatment of asthma, including bronchodilator inhalers paired with anti-inflammatory drugs (i.e., inhaled corticosteroids) [22].

Limitations of the study

There are several limitations within this study that must be highlighted. First, the retrospective collection of data is known to pose the risk of missing, or potentially incorrect information, whilst also being affected by selection and recall bias [23]. This was the case with our analysis, with certain parameters missing, e.g., spirometry results, lifestyle factors, and relevant medical history. Moreover, certain patients may have been lost to follow-up and cannot be ascertained, hence leading to additional bias. Furthermore, the limited timeframe of retrospective data collection from January 2022 to April 2023 relies on the assumption that the retrospective trends will not change - however, long-term effects of COVID-19 and emerging novel variants may result in unexpected differences within future results. Additionally, the smaller sample size may not be entirely representative or generalizable of the local or global population, thus introducing some heterogeneity within the results of this study. Lastly, the exclusion of coincidental development of adult-onset asthma cannot be enforced on the post-COVID cohort; therefore, the cluster of symptoms cannot be fully ascertained to be caused by COVID-19 infection. Ultimately, further comprehensive and robust studies are essential in order to validate our conclusion.

## Conclusions

In conclusion, the post-COVID asthma-like symptoms including cough, chest tightness, and SOB were observed to be statistically common with the cohort of known asthmatic patients, indicating the possibility of the development of a new post-COVID syndrome that mimics the clinical presentation of asthma. Moreover, with respect to the investigative parameters evaluated, there were significant similarities observed within the serum IgE and spirometry results between both cohorts. Furthermore, all patients with post-COVID asthma-like symptoms were completely relieved of their symptoms after bronchodilator therapy for a short-term duration, indicating the potential therapeutic benefit of using targeted therapy, similar to that used within asthmatic patients. However, in order to arrive at a valid conclusion, future comprehensive studies are recommended in order to explore the exact pathophysiology of these symptoms, and effective long-term management strategies in order to provide symptomatic relief for such patients.
